# Respiratory Depression in Non-Operating Room Anesthesia: An Overview

**DOI:** 10.3390/jcm14134528

**Published:** 2025-06-26

**Authors:** Isabel E. Royz, Nicholas B. Clevenger, Andrew Bochenek, Andrew R. Locke, Steven B. Greenberg

**Affiliations:** 1 Department of Anesthesiology, Critical Care, and Pain Medicine, Endeavor Health, Evanston, IL 60201, USA; isabelroyz15@gmail.com (I.E.R.); nickclev11@gmail.com (N.B.C.); abochenek@northshore.org (A.B.); lockeandrewr@gmail.com (A.R.L.); 2 Department of Anesthesiology and Critical Care, Pritzker School of Medicine, University of Chicago, Chicago, IL 60637, USA

**Keywords:** anesthesia, non-operating room anesthesia, perioperative care, adverse respiratory events, respiratory monitoring

## Abstract

Non-operating room anesthesia (NORA) is a rapidly growing domain for anesthesia professionals due to advances in procedural technology and increased emphasis on patient comfort. The majority of these procedures are conducted under monitored anesthesia care (MAC) where patients receive varying levels of sedation. Analysis of the Anesthesia Closed Claims database suggests that adverse respiratory events continue to be the main cause of morbidity and mortality in patients undergoing NORA procedures. Most NORA claims occurred under MAC, with oversedation leading to respiratory depression coupled with inadequate monitoring making up the majority of claims. The American Society of Anesthesiologists (ASA) has released standards of pre-anesthesia, intraoperative monitoring, and post-anesthesia care, which apply to all anesthetizing locations including NORA. The ASA has also made recommendations in a statement on NORA to promote patient safety. Evidence suggests that patient characteristics, monitoring tools, physical constraints, and team familiarity play a role in the risk for adverse respiratory events. Future studies are required to further understand the challenges specific to NORA locations.

## 1. Introduction

Non-operating room anesthesia (NORA) is a rapidly growing area of anesthesia provision of care, occurring in hospital locations outside of the operating room (OR) and office-based settings. The most recent literature regarding case load from the National Anesthesia Clinical Outcomes Registry suggests that NORA cases have increased from 28.3% in 2010 to 35.9% in 2014, and are projected to match or exceed the number of OR cases in the future [1,2,3,4,5,6,7,8]. Although no specific data have been updated since 2014, the percentage of NORA cases has likely already increased. An updated analysis of NORA case load should be performed to understand the evolution of this field in recent years. Growth in NORA can be attributed to multiple reasons, ranging from an increase in recommended screening procedures to technological advancements. The fields that commonly incorporate anesthesia services include gastroenterology (GI), pulmonology, cardiology, and interventional radiology. For the purpose of this discussion, NORA will focus on areas of the hospital that provide procedural care outside of the operating room, excluding obstetrics and office-based settings. In the past, proceduralists or nurses would provide conscious sedation while monitoring the patient [9,10,11]. However, with technological advances and a greater emphasis on patient comfort through the use of propofol, anesthesia providers have been involved to provide these higher levels of anesthesia [2,3,6,9,12,13,14,15,16]. Furthermore, patients undergoing these procedures can have complicated medical histories and can be part of the geriatric population that may require extra surveillance [1,2,11,13,14,17,18,19,20,21,22,23,24]. These developments have led to a growing demand for anesthesia providers to deliver safe care for patients in NORA locations.

From a healthcare system perspective, NORA procedures are cost-effective for hospitals and can streamline care by potentially decreasing the length of stay with less invasive procedures and improving patient satisfaction [18,19,25,26]. One reason for the quicker recovery is that NORA procedures typically utilize monitored anesthesia care (MAC) as opposed to general anesthesia (GA) [15,17,27,28,29]. According to the American Society of Anesthesiologists (ASA), MAC is a type of anesthesia service in which an anesthesia professional continually monitors and supports the patient’s vital functions and diagnoses and treats clinical problems that occur. This is typically under the guise of administering sedative, anxiolytic, or analgesic medications if needed but not absolutely necessary [30]. When using GA, comparatively, patients are fully unconscious and often times require assisted positive pressure ventilation through an endotracheal tube, mask, or subglottic device [6,25,31]. Patients under GA may have longer recovery periods in the post-anesthetic care unit (PACU) due to their deeper anesthetic depth and are often at risk for nausea and vomiting when inhaled anesthetics are used [32]. The greater use of MAC in non-operating room procedures may facilitate faster procedure times, a reduction in the use of the PACU or bypass of the PACU into a secondary recovery area, decreased risk of airway injury, and decreased side effects from inhaled agents [25,32,33].

Despite the discussed advantages of NORA procedures, they may invoke inherent risks that can be compared to operating room procedures [13,34]. When evaluating a 2006 Closed Claims analysis, it was reported that patients in NORA locations experienced greater severity of injuries than patients undergoing OR procedures [17]. Similar trends were observed in the Closed Claims review from 2017 by Woodward et al., where death and permanent brain damage continued to occur at higher rates in NORA than in the OR [22]. These studies among others have reported that majority of these claims stem from adverse respiratory events [7,14,17,18,22,27,32,35]. Most claims highlighted the use of MAC, where inadequate monitoring could have been the major issue resulting in these poor outcomes [17,18,22,23].

In addition to the clinical risks that may occur with NORA, another contributing factor for adverse outcomes is that NORA locations have unique infrastructural differences when compared to the OR that pose challenges to anesthesia professionals. This topic will be discussed in Section 6.1: Physical Constraints.

With prior studies and Closed Claims reports demonstrating that respiratory depression is one of the leading causes of morbidity and mortality in NORA, it is important to discuss the associated causes, implications, and prevention techniques by utilizing appropriate monitoring tools and setting cultural standards. The current review will highlight the causes of adverse respiratory events among patients undergoing hospital-based NORA and the current and potential preventative measures using the guiding documents in Table 1.

## 2. Adverse Respiratory Events

Despite the known concern for adverse respiratory events in NORA locations, they continue to be the main cause for morbidity and mortality. The main events that patients may experience when undergoing NORA are hypoxemia and/or hypercapnia/hypoventilation. Hypoxemia refers to inadequate oxygenation and an inability to maintain appropriate blood oxygen levels [13,40]. Hypercapnia refers to inadequate ventilation in which the body is unable to eliminate carbon dioxide (CO_2_) [13,40]. Most studies that analyzed national databases have found that these adverse respiratory outcomes can be attributed to oversedation in the setting of inadequate monitoring [7,15,17,18,22,23,41]. Oversedation coupled with procedural demands that create difficulty in providing oxygenation/ventilation for a patient, such as a shared airway with the proceduralist, further increases the risk of detrimental respiratory events.

## 3. Standards and Recommendations for NORA

With the continued growth of NORA, the ASA Committee of Surgical and Procedural Anesthesia released an updated *Statement on Nonoperating Room Anesthesia Services* in 2023 to provide recommendations for safe conduct of NORA [36]. These are foundations to ensure safety in the NORA setting; however, they can be expanded upon by the anesthesia professional at their clinical discretion. This statement breaks down leadership and oversight structure of NORA care into several domains, with an overall recommendation to make it similar to OR-based anesthesia. In the statement, there is an emphasis placed on having emergency or resuscitative equipment readily available, quality improvement efforts, efficient case scheduling, availability of appropriate personnel, outlined hospital-system based protocols (i.e., sterilization of equipment, access to the patient’s medical record, operational and quality metrics, process of activating a code/rapid response, etc.), and more [36]. It is recommended that the lead anesthesia professional for each NORA location is involved in creating appropriate policies and standard operating procedures as it adheres to the ASA standards of care [36]. The following sections will discuss the most recent pre-, intra-, and post-procedural standards of care as outlined by the ASA.

### 3.1. Pre-Procedural Standards

According to the 2020 *ASA Basic Standards for Preanesthesia Care*, all patients who receive anesthesia care should receive a pre-anesthetic assessment by an anesthesia professional [39]. This assessment will help determine the medical status of the patient to thus prepare an appropriate anesthesia care plan [39]. According to the ASA, six actions should be executed during a pre-anesthesia assessment as listed in Table 2. Further information on patient selection for NORA will be discussed in Section 4.1: Patient Selection. Additionally, a focused assessment that keeps all of the NORA specific challenges in mind, as discussed in Section 6: Cultural Considerations in NORA, may help further optimize safety during anesthetic.

### 3.2. Intra-Procedural Standards

Following the completion of a pre-anesthetic evaluation, the patient will begin to receive anesthesia for their procedure. The 2020 *ASA Standards for Basic Anesthetic Monitoring* discuss what monitors should be used when providing any type of anesthetic (i.e., GA or MAC), aside from emergent situations (Table 3) [38]. Within NORA, a patient’s status can change at any time as they receive anesthesia. Therefore, a qualified anesthesia provider has to be present in the room when MAC or GA are being delivered for patient monitoring [38]. If an anesthesia professional is unable to physically be near the patient given the constraints of the procedure (i.e., radiation or positioning), there has to be an alternative method of monitoring available [38]. Additionally, the patient’s oxygenation, ventilation, circulation, and temperature should be evaluated [38]. Monitoring tools such as an anesthesia machine in the case of GA, or pulse oximetry, capnography or capnometry, electrocardiogram and temperature probes should be incorporated [38]. Qualitative monitoring, such as chest excursion or skin coloration, can also be used to monitor the above stated vitals [38]. Section 5: Monitoring in Non-Operating Room Anesthesia will further discuss information on respiratory monitoring devices. While this section will only focus on respiratory monitoring, as per the ASA standards, circulatory and thermal monitoring is required for each procedure [38].

### 3.3. Post-Procedural Standards

Once the procedure concludes, the patient will begin to wake up from anesthesia. The 2024 ASA *Standards for Postanesthesia Care* provide direction as to how post-anesthesia care should be delivered (Table 4) [37]. Typically, patients undergoing general anesthesia and certain MAC cases that require a higher level of monitoring and nursing care will go to the PACU. This PACU area should meet the facility’s requirements and medical care should be performed under the policies and procedures of the Department of Anesthesiology [37]. When the patient is being transferred from the procedural room to the recovery area, they should be accompanied and monitored by someone from the anesthesia care team to ensure safe transport and disclosure of pertinent history and case details [37]. In the PACU, the patient should be monitored for oxygenation, ventilation, circulation, level of consciousness, and temperature throughout the recovery period [37]. Once a patient has met the appropriate PACU discharge criteria determined by the designated anesthesia professional, they may be discharged to their next location [37]. Based on the customized anesthesia plan set forth by the multidisciplinary care group, including the anesthesia professional and procedural team, certain patients who undergo MAC may be able to bypass the PACU to a step-down recovery area [25,33,42,43]. Further information on post-anesthesia care for patients following NORA will be discussed in Section 7: Post-Operative Care Recommendations.

## 4. Patient Population Considerations

The ASA Physical Status Class is a five-tier system used to assess a patient’s pre-anesthetic medical co-morbidities, with a higher class denoting more severe co-morbidities [44]. In a trend analysis, Nagrebetsky et al. determined that the percentage of NORA patients with ASA Physical Status Class III-V was higher (37.6%) than the traditional OR group of patients (33.0%) [1]. Given that a greater percentage of patient’s receiving NORA fall into the elevated ASA Physical Status Class, there is concern that these patients are at a greater risk for experiencing complications throughout the procedure [13,26]. The higher percentage of greater ASA class status patients in NORA may be due to the fact that these patients were deemed too high risk for operative procedures.

### 4.1. Patient Selection

The 2022 Anesthesia Patient Safety Foundation (APSF) Stoelting Conference released consensus recommendations from a multidisciplinary group of experts on the safe practice of NORA, which include patient selection [4]. The statements recommend providing specialized preprocedural evaluation for unique comorbidities and determining if the planned procedure and location is suitable for the patient [4]. Furthermore, Georgiadis et al. suggest incorporating an anesthetic clinic evaluation when possible prior to the day of the procedure to determine a patient’s candidacy for NORA and, if necessary, allow for earlier optimization of their pre-existing conditions [2]. When evaluating a patient for a procedure, it is recommended to pay attention to the patient’s airway and their ability to protect it, especially when planning for non-invasive ventilation [2,15,35,43,45]. As NORA continues to expand, it is important to include the anesthesia team to facilitate appropriate patient selection and optimization prior to the procedure.

### 4.2. Considerations for Complex Patients in NORA Care

The growing number of unique populations including geriatric, obese, and medically complex patients with multiple comorbidities increases the need for the NORA team to be aware of the heightened susceptibility of these patients to develop respiratory events. Providers participating in these patients’ care should be able to manage these complex situations in a way that maximizes safety and contributes to the well-being of the patient.

#### 4.2.1. Geriatric Population

Nagrebetsky et al. found that the mean age of patients undergoing NORA procedures was 3.5 years greater than patients undergoing traditional OR procedures [1]. The geriatric population may be at greater risk for respiratory complications in the surgical setting due to the natural ageing process and comorbidities. With age, structural changes may lead to decreased chest wall compliance and respiratory muscle strength [46,47], increased lung compliance [48], increased residual volume [47], and increased respiratory system resistance [49]. In addition, the geriatric group may be more sensitive to anesthetic agents and opioids due to differences in pharmacokinetics and pharmacodynamics [50]. Reduced renal and hepatic clearance coupled with an increase in volume of distribution for lipophilic drugs may lead to increased sensitivity and prolonged therapeutic effects of anesthetic medications including propofol, benzodiazepines, and lipophilic opioids [51]. These age-related changes give rise to the potential for respiratory events [52,53]. This further demonstrates the need for a thorough preoperative evaluation and increased vigilance throughout the perioperative period to ensure safe, individualized care.

#### 4.2.2. Obese Population

Obese patients (body mass index (BMI) ≥ 30 kg/m^2^) [54] are also at an increased risk for respiratory complications in the operative setting [55]. Altered respiratory mechanics are seen due to excess adipose tissue deposition on the chest wall, abdomen, and upper airway. With increasing BMI, there is a decrease in functional residual capacity (FRC), respiratory system compliance, and oxygenation index. There is an increase in the work of breathing and resistance of the total respiratory system [56,57]. Obesity alters the pharmacokinetics and pharmacodynamics of most drugs. Drug dosing based on ideal body weight may lead to underdosing, while dosing based on actual body weight (ABW) may lead to overdosing or a prolonged therapeutic effect. This overdosing of sedatives and analgesics can lead to an increased risk for hypoventilation and hypoxemia. One proposed solution is to dose based on calculated lean body weight (LBW) followed by titration to desired pharmacologic effects [58]. Several authors have worked to formulate an equation that could be used to accurately calculate LBW [59,60].

#### 4.2.3. Obstructive Sleep Apnea

Obesity is a known risk factor for obstructive sleep apnea (OSA). With approximately 40% of adults in the United States meeting the obesity criteria [61], careful preoperative screening and anesthetic planning should be conducted for these patients [62]. These include but are not limited to a review of sleep studies and the use of continuous positive airway pressure (CPAP) or oral appliances during sedation [62]. Obstructive sleep apnea is an extremely common breathing disorder in the United States that affects nearly 34% of men and 17% of women [63]. OSA patients have recurrent episodes of partial or complete obstruction of the upper airway due to collapse. Increasing the depth of propofol anesthesia is associated with an increase in upper airway collapsibility and an increased risk of hypoxemia and postoperative complications for this population [64]. Anesthetic medications including propofol, benzodiazepines, and opioids have also been associated with impairment of the arousal response; a defense mechanism that helps overcome airway obstruction.

## 5. Monitoring in Non-Operating Room Anesthesia

### 5.1. Current Practices and Relevance

More than half of the reported NORA adverse respiratory event claims are attributed to inadequate monitoring [5,7,12,14,17,18,22,23,27,28,29,65]. Given the risk of losing an airway due to oversedation when undergoing MAC, it is important to address this issue. While other risks still exist, this section will specifically focus on monitoring to detect and prevent respiratory depression.

### 5.2. Oxygenation Monitoring

During many NORA cases where anesthesia professionals apply MAC, the pulse oximeter is used alone and provides a quantitative measure of the oxygen saturation (SpO_2_) in a patient’s red blood cells [66]. However, in 2021, the Food and Drug Administration issued a safety communication that lists several limitations of the pulse oximeter during monitoring of patients with different skin pigmentations, skin temperatures, and fingernail polish [67]. Such limitations can lead to inaccurate measures of oxygenation, thus emphasizing the need for the continued development of appropriate algorithms in pulse oximeter monitors to account for these variables. However, some evidence suggests that pulse oximetry accuracy may not differ significantly across different skin tones [68]. As a result, this issue still warrants further investigation to improve accuracy across diverse populations [69]. Nevertheless, continuous vigilance is key and when an issue arises, it is important for providers to promptly intervene by reducing sedation levels or taking corrective action to prevent the progression of respiratory related issues. The pulse oximeter is also not as accurate in detecting hypoventilation or apnea, especially when using supplemental oxygen [70,71,72]. The threshold alarm settings can also cause delayed detection or false positives, depending on their configuration, which lowers the reliability of early warning systems [73]. Despite its limitations pulse oximetry is widely used because it is non-invasive, well-tolerated by patients, readily available, and inexpensive [74].

### 5.3. Ventilation Monitoring

As hypoventilation, apnea, and decreased respiratory rate are indicators of drug-induced respiratory impairment, ventilation monitoring is essential in preventing respiratory depression [75]. Qualitative assessment methods such as direct observation of chest rise and auscultating breath sounds are commonly used but are insufficient on their own to assess ventilation and should be supplemented with additional quantitative monitoring technologies, such as capnography [13]. By providing a continuous waveform of the expired carbon dioxide, capnography is recommended for all anesthetized patients to help minimize the risk of an anesthesia adverse event from inadequate ventilation [76]. The use of capnography compared to pulse oximetry alone has been shown to detect ventilatory depression earlier [77] and reduced the occurrence of severe hypoxemia by 16% [78]. A different study by Soto et al. found that when using capnography, all apneic episodes were detected in patients under MAC, even with varying amounts of supplemental oxygen [70].

However, capnography does have limitations. In endoscopic operations, the end-tidal CO_2_ levels may be elevated unrelated to ventilation due to the entrainment of CO_2_ from insufflation [79]. Patient tolerance is another concern as nasal sampling lines can become unpleasant or displaced during treatment [80]. Despite the limitations and the costliness of the monitor itself, capnography remains a valuable tool for detecting respiratory depression and is required by the ASA Standards for Basic Anesthetic Monitoring [36,38].

### 5.4. Modalities for Reduction in Airway Obstruction

There are additional devices that may be used to help mitigate the occurrence of airway obstruction, specifically in procedures that require a shared airway. These include a nasal CPAP and high flow nasal cannula (HFNC). Both of these are non-invasive ventilation devices that provide positive airway pressure to maintain an open airway in patients. The nasal CPAP optimizes oxygenation by reducing the work of breathing [81,82]. The preprocedural optimization with a CPAP of patients, specifically with obstructive sleep apnea, may reduce postoperative cardiovascular complications and lengths of stay [83]. Some limitations of the nasal CPAP may be patient tolerance or nasal discomfort due to dry air [81,82].

The HFNC delivers humidified, heated oxygen at greater flow rates compared to conventional supplemental oxygen, improves ventilation efficiency, and enhances oxygen delivery [57,81,84]. A systematic review by Tao et al., found that the use of HFNC in NORA GI procedures resulted in a slight improvement in oxygenation, need for airway intervention, and procedure interruptions compared to using conventional oxygen therapy [85]. The humidified air in the HFNC removes the potential nasal discomfort caused by the nasal CPAP; however, it is a more complex, expensive device whose equipment may take up already limited space [57,81,84]. Both devices also carry the risk of limited efficacy given there is not a tight seal around the entire airway passage (i.e., breathing through an open mouth) [82,84]. Despite these limitations, both have been shown to help patients experiencing respiratory depression [81,82,83,84].

### 5.5. Future Direction

Advancements in technology are paving the way for enhanced monitoring of respiratory function during anesthesia. There are emerging wearable monitoring technologies in the field of anesthesia that could enhance traditional perioperative respiratory monitoring [86]. These devices look to integrate advanced biosensors that can detect parameters like oxygen saturation, respiratory rate, and even subtle changes in chest and abdominal movement [87,88,89]. Non-invasive vital parameter monitoring approaches have the potential to significantly improve the management and monitoring of respiratory depression in NORA situations. Furthermore, integrating AI into monitoring systems can make predictive analytics possible and improve early identification for patients susceptible to respiratory depression [90]. For example, the PRODIGY trial introduced a risk prediction tool that utilizes continuous capnography and oximetry data to identify patients at higher risk of opioid induced respiratory depression, allowing for faster interventions [91]. Integrating technologies such as AI and wearable monitoring tools could allow for more precise and individualized patient care, enhancing safety in NORA locations.

## 6. Cultural Considerations in NORA

A recent study by Schroeck et al. found that anesthesia professionals experience increased levels of stress and anxiety and a decreased perception of safety when working in NORA environments compared to the OR [92]. There are several challenges that are unique to the NORA environment that could play a role in increasing stress levels for providers and thus put patients at risk. Table 5 summarizes the challenges that anesthesia professionals may face in NORA.

### 6.1. Physical Constraints

One challenge is the physical differences between NORA and OR locations. Several papers have listed the remote location of NORA procedure rooms, either from each other or from centralized OR locations, as a challenge in providing safe care for patients [4,14,15,19,21,22,26,28,93]. These remote areas could create challenges in accessing equipment, medication, or anesthesia-trained personnel in emergency situations. Additionally, Nagrebetsky et al. found that NORA cases were more likely to start after hours (17:01–06:59) compared to OR cases [1]. Non-daytime working hours coupled with the remote NORA locations may limit the availability of additional help that anesthesia professionals may need to safely provide anesthesia to patients.

**Figure 1:**
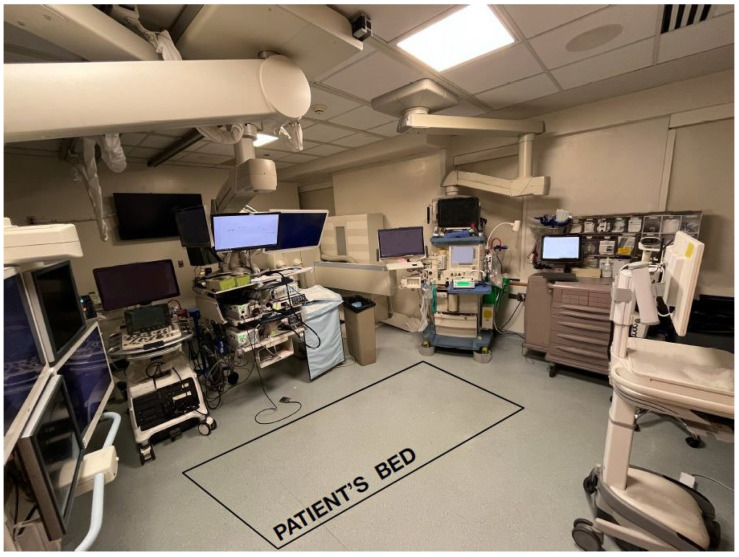
A GI procedure room is depicted where patients may receive MAC or GA while undergoing endoscopy, colonoscopy and endoscopic retrograde cholangiopancreatography (ERCP). This procedural room can be a challenging place for anesthesia professional patient care as the anesthesia equipment is often times in the corner, the lights are dimmed throughout the procedure, the patient is often times facing away from the anesthesia professional in the prone position and the airway is often shared with the proceduralist.

There are further differences between a NORA procedural room set-up when compared to the OR, as denoted in Figure 1. Along with the limited working space, it may be difficult for the anesthesia provider to visualize the patient due to the procedural needs (i.e., having a shared airway, patient positioning, dull lighting, imaging machinery, etc.) [2,5,14,19,21,26,28,35]. Having limited visualization of the patient can impose challenges in monitoring a patient’s respiratory status (i.e., skin coloration, chest excursion, etc.) [2,9,28].

One reason for the lack of appropriate anesthesia work space in NORA locations may be attributed to the fact that the procedural rooms are often times built in the absence of anesthesia professional input [2,4,5,9,11,14,19,21,22,26,28,34,65,93].

Therefore, the 2023 ASA *Statement on Nonoperating Room Anesthesia Services* recommends including the Director of Anesthesia Services or another administrative designee to help design, re-design, or re-purpose procedure rooms to account for the safe delivery of anesthesia care [36]. Furthermore, they state that NORA rooms should either be close to the centralized OR or in close proximity to each other to increase safety and efficiency of procedures [36]. Additionally, each NORA room should have adequate sources of oxygen, suction, waste gas removal, electrical outlets, illumination, and space [36]. One anesthesia group suggested that NORA rooms should have at least 84 square feet of space for anesthesia services to account for the anesthesia machine, medication and equipment carts, anesthesia chair, and poles for IV bags [94]. Optimizing NORA scheduling may occur by integrating it into the main OR case scheduling system [36]. Given the increased risk for adverse respiratory events, it is recommended that NORA environments are well-equipped to either prevent these events from occurring or be prepared to handle them.

### 6.2. Team Familiarity

Along with optimizing the location, it is important for patient safety to ensure that all members of the NORA team can communicate effectively with one another. One way to promote familiarity is to have open communication, or the unrestricted sharing of information, amongst the healthcare team throughout the perioperative period [4,6,9,19,26,28,95,96]. This allows everyone to have a better understanding of the demands of the procedure and the patients’ risks. Additionally, incorporating a closed-loop communication system could be beneficial in ensuring accurate understanding and enactment of plans throughout the procedure by verbalizing an idea or request, repeating it back, and confirming it [97].

Chang and Dudley also recommend utilizing a time-out checklist unique to NORA environments, given there is not currently a standardized one [98]. While this checklist is similar to one that is used in the OR, they recommend to use it prior to the induction of anesthesia and to include the entire healthcare team to ensure that everyone is aware of the demands of the procedure and patient needs [98]. The literature generally has supported creating and enforcing checklists in NORA environments [65,99]. These checklists may include identification of the patient, procedural needs (i.e., positioning), anesthetic plan, team members, emergency responses, and post-procedure plans [65,98,99]. However, an appropriate balance must be considered among all stakeholders between implementation of structured NORA specific checklists and checklist fatigue, which entails being disengaged from the checklists due to the sheer number and content of them [99,100,101]. This is where customization of these checklists by the key stakeholders in NORA care may facilitate improved compliance among the healthcare professionals who work in NORA locations.

Finally, it is important to appropriately train non-anesthesia personnel who work in NORA environments with regard to anesthesia specific concerns and how to respond to emergency events. Given that staff working in NORA environments may not be familiar with specific OR procedures that anesthesia professionals are familiar with, or working with anesthetized patients, it is important to implement continuous learning efforts and training for non-anesthesia personnel [19,36]. Anesthesia professionals and non-anesthesia staff alike may become more comfortable in navigating acute adverse events after exposing them to simulation exercises that incorporate NORA location safety issues [3,4,6,15,27,41,99]. These may include dealing with acute hypoxemia, hypoventilation, cardiac or respiratory arrest, or a lack of functional equipment.

## 7. Post-Operative Care Considerations

Following an outpatient procedure, a patient may either enter Phase I (i.e., PACU) or go directly to Phase II (i.e., Ambulatory Surgery Unit) [102,103]. Patients are able to bypass Phase I based on their level of recovery and Post-Anesthetic Recovery Score (PARS or Modified Aldrete Scoring System) at the end of the procedure [102,104]. The PARS score assigns points for the following criteria: level of consciousness, respiration, circulation, oxygen saturation, and level of activity [103]. Given the short-acting anesthetic medication that patients receive when undergoing MAC and the post-procedural plan between the anesthesia and procedural team, patients are typically able to bypass Phase I and go directly to Phase II [25,33,42,43]. In Phase II, patients are assessed for home readiness using the Post Anesthetic Discharge Scoring System (PADS) [4,14,102,103,105]. In this area, patients typically undergo periodic monitoring by nurses who may be overseeing multiple patients at a time [42,106]. Patients are prepared for discharge with a responsible adult by receiving post-operative and follow-up care instructions [4,13,42,102,103,107,108]. Throughout the recovery process, there should be clear documentation by a responsible anesthesia professional or nurse to demonstrate that the patient has met appropriate criteria [4,9,12,14,26,103]. Table 6 lists some of the recovery criteria that are evaluated during Phase I and Phase II.

## 8. Conclusions

Respiratory depression in NORA locations remains a significant patient safety concern. The lack of standardization, aging patient population, remote locations from the operating room, and unfamiliarity among NORA team members are all contributing factors to the high incidence of respiratory complications in NORA. The most recent ASA guidelines and the 2022 APSF Consensus recommendations are a major step to providing guidance and opening the dialogue amongst NORA providers to enhance the patient safety issues at hand [4,36,37,38,39]. Further studies are required to address the challenges due to space constraints, team unfamiliarity, and monitoring specific to NORA locations. Additionally, these concerns should be addressed in a multidisciplinary manner with anesthesia representation in the remodeling and new construction of NORA locations. However, one thing is clear: anesthesia professionals are a key component to the reduction in perioperative respiratory adverse events specific to NORA locations.

## Figures and Tables

**Table 1:** List of guiding documents [4,19,36,37,38,39].

Key Referenced Documents
1. ASA Statement on Nonoperating Room Anesthesia Services
2. ASA Basic Standards for Preanesthesia Care
3. ASA Standards for Basic Anesthetic Monitoring
4. ASA Standards for Postanesthesia Care
5. APSF Newsletter: Safety in Non-Operating Room Anesthesia (NORA)
6. Consensus Recommendations for the Safe Conduct of Nonoperating Room Anesthesia: A Meeting Report From the 2022 Stoelting Conference of the Anesthesia Patient Safety Foundation.

**Table 2:** Aspects of NORA pre-anesthesia assessment [19,39].

**Aspects of the Pre-Anesthesia Assessment**	
1. Review of past medical records	2. Interviewing the patient to learn about their past medical history, anesthesia experience, and carrying out a focused physical exam
3. Performing appropriate testing and consultations	4. Ordering appropriate medications
5. Obtaining consent for anesthesia	6. Accurately documenting the encounter in the chart
**Additional Perspectives from the APSF**	
7. Pre-procedure timeout per The Joint Commission Universal Protocol	8. Assessment of fall and venous thromboembolism risk including appropriate treatment

**Table 3:** Aspects of NORA intra-operative anesthesia management [38].

Aspects of Intra-Operative Anesthesia Management	
1. Qualified anesthesia personnel present throughout anesthesia care	2. Continual monitoring of oxygenation, ventilation, circulation, and temperature by way of pulse oximetry, capnography or capnometry, electrocardiogram, and temperature probes
3. Qualitative monitoring using chest excursion or skin coloration	4. Established formal system in place to call for assistance and designate personnel to respond and transport the patient

**Table 4:** Aspects of NORA post-operative anesthesia management [19,37].

Aspects of Post-Operative Anesthesia Management	
1. Patients should receive appropriate post-anesthesia care in PACU or area that provides equivalent support	2. Patient should be accompanied by member of anesthesia team during transport and continually evaluated and treated with appropriate monitoring and support
3. Upon arrival to the PACU or designated area, patient should be re-evaluated and verbal report given by member of anesthesia team to nurse responsible for receiving patient	4. Physician responsible for patient discharge from PACU or other designated area

**Table 5:** Summary of challenges anesthesia professionals face in NORA environments.

NORA Challenges for Anesthesia Professionals	
Remote location	After-hour case starts
Limited anesthesia workspace	Rooms with inadequate lighting
Shared airway	Patient positioning
Imaging machinery	Lack of access to equipment, medications, and anesthesia trained personnel
Unfamiliar healthcare team	Lack of standardized time-out checklist

**Table 6:** Examples of recovery criteria that patients may need to meet to be discharged from Phase I and Phase II [13,26,102,103,104,105].

**Phase I Recovery Criteria**	
Hemodynamic Stability	Level of Consciousness
Voluntary Physical Activity	Respiratory Stability
Maintaining Oxygen Saturation	Minimal Post-Operative Nausea/Vomiting/Pain
**Phase II Recovery Criteria**	
Stable Vital Signs	Ambulating at Baseline without Dizziness
Minimal-No Nausea/Vomiting/Pain	Minimal Post-Operative Bleeding

## Abbreviations

ABW	Actual Body Weight
APSF	Anesthesia Patient Safety Foundation
ASA	American Society of Anesthesia
BMI	Body Mass Index
CO_2_	Carbon Dioxide
CPAP	Continuous Positive Airway Pressure
GA	General Anesthesia
GI	Gastrointestinal/Gastroenterology
HFNC	High Flow Nasal Cannula
LBW	Lean Body Weight
MAC	Monitored Anesthesia Care
NORA	Non-Operating Room Anesthesia
OR	Operating Room
OSA	Obstructive Sleep Apnea
PACU	Post Anesthesia Care Unit
PADS	Post-Anesthetic Discharge Score
PARS	Post-Anesthetic Recovery Score
SpO_2_	Oxygen Saturation
